# From Peer Support to Program Supervision: Qualitative Insights on WhatsApp as Informal Digital Infrastructure for Community Health Workers and Public Health Officers in an Indian High-Priority Aspirational District

**DOI:** 10.3390/healthcare13172223

**Published:** 2025-09-05

**Authors:** Anshuman Thakur, Reshmi Bhageerathy, Prasanna Mithra, Varalakshmi Chandra Sekaran, Shuba Kumar

**Affiliations:** 1 Department of Health Information Management, Manipal College of Health Professions (MCHP), Manipal Academy of Higher Education (MAHE), Manipal 576104, Karnataka, India; anshuman.mpl@learner.manipal.edu; 2 Community Medicine, Kasturba Medical College, Mangalore, Manipal Academy of Higher Education (MAHE), Mangalore 575001, Karnataka, India; prasanna.mithra@manipal.edu; 3 Health Policy, Prasanna School of Public Health (PSPH), Manipal Academy of Higher Education (MAHE), Manipal 576104, Karnataka, India; varalakshmi.cs@manipal.edu; 4 Samarth, Chennai 600004, Tamil Nadu, India; shubakumar@samarthngo.org

**Keywords:** WhatsApp, community health workers (CHWs), digital health, program supervision, mHealth, health communication, digital equity, qualitative research, aspirational district, India

## Abstract

**Background**: In low-resource health systems, official mHealth platforms often face usability and infrastructure barriers. In India, Community Health Workers (CHWs) and their supervisors have pragmatically turned to WhatsApp as an informal digital infrastructure. While widely adopted, its dual role as both a support system and a source of burden remains underexplored. **Aim**: To examine the patterns and purposes of WhatsApp use among CHWs and block-level supervisors; to identify perceived benefits, barriers, and risks; and to assess its influence on workflow, power relations, digital equity, and program outcomes in an Indian Aspirational District. **Methods**: We conducted a qualitative study between June and December 2023 in Muzaffarpur, Bihar, India. Data comprised 32 in-depth interviews and six focus group discussions with CHWs (Anganwadi Workers, ASHAs, ANMs) and block-level public health officers (total participants *n* = 81). We used reflexive thematic analysis following Braun and Clarke’s approach; reporting adhered to the COREQ guideline. **Results**: WhatsApp emerged as a de facto digital backbone for real-time communication, peer support, and program supervision, often perceived as more usable than official applications. Its informal adoption also created a triple burden: digital fatigue from information overload and blurred work–life boundaries; duplication of reporting across WhatsApp and official portals; and systemic inequities that disadvantaged older or less digitally literate CHWs, with risks of surveillance creep and data privacy breaches. **Conclusion:** WhatsApp simultaneously enables coordination and imposes workload and equity costs on India’s frontline workforce. Without formal policy and governance, this user-driven adaptation risks widening digital divides and accelerating burnout. We recommend clear protocols on purpose-limited use, investments in equitable digital capability and devices, and safeguards that protect worker well-being and data privacy.

## 1. Introduction

Digital transformation is redefining the landscape of public health delivery, particularly in low- and middle-income countries (LMICs) like India, where the rapid adoption of mobile technologies holds promise for bridging gaps in health communication, program supervision, and data management [1,2]. To ensure ground-level penetration of these technologies and program implementation, Community Health Workers (CHWs), including Anganwadi Workers (AWWs), Accredited Social Health Activists (ASHAs), and Auxiliary Nurse Midwives (ANMs), are pivotal for last-mile delivery, particularly in low-resource settings and India’s ‘Aspirational Districts’ that lag on composite development indicators [3,4,5,6,7].

National digital health initiatives such as the Ayushman Bharat Digital Mission and platforms like ANM Online (ANMOL) and the POSHAN Tracker have sought to streamline service delivery, reporting, and beneficiary tracking [8,9,10]. However, despite strong policy momentum, field-level implementation of digital health solutions is often hindered by a complex array of operational barriers. These include inconsistent internet connectivity, device malfunction, frequent power outages, limited technical support, and a lack of context-specific digital literacy among CHWs [11,12,13,14].

These challenges are most acute in India’s aspirational districts, regions targeted explicitly by the Government of India for intensive intervention due to their lagging socioeconomic and health indicators [15,16]. In these settings, CHWs routinely contend with dual reporting requirements, balancing paper registers with digital data entry, and often receive limited training or ongoing mentorship for new technologies [11,17,18,19]. The result is a widening gap between the policy intent of digital transformation and the operational realities experienced by frontline workers, frequently leading to increased administrative burden, reduced programmatic efficiency, and declining motivation [13,14,19,20,21,22,23,24].

Beyond infrastructure, the digital divide is compounded by social determinants such as age, education, gender, and geographic remoteness, which differentially shape CHWs’ access to and engagement with digital tools [25,26]. Several studies report the challenges of formal digital systems, especially in low-resource settings and among CHWs [19,23,27]. In response, many frontline workers have gravitated towards informal, widely accessible platforms, particularly WhatsApp, as alternative channels for information sharing, supervision, and peer support [28,29].

WhatsApp, with its ease of use, real-time communication, and low data requirements, has emerged as a “good enough” infrastructure for supporting health program operations in resource-limited environments [29,30,31]. Its widespread adoption among Indian CHWs is not simply a matter of convenience but reflects a pragmatic adaptation to systemic barriers in the official digital health ecosystem [12,32,33]. During the COVID-19 pandemic, WhatsApp groups served as critical conduits for disseminating urgent instructions, facilitating virtual supervision, coordinating community events, and providing psychosocial support to isolated workers [31,34,35].

Nevertheless, the informal integration of WhatsApp into routine program management is not without risks. Emerging evidence highlights challenges such as information overload, duplicative reporting, work-life boundary erosion, heightened digital surveillance, and increased stress among CHWs [29,35,36,37,38,39]. More troubling are concerns regarding data privacy, confidentiality, and the ethical exchange of sensitive beneficiary information on commercial, non-government platforms [40,41,42]. For many, these challenges are further amplified by the lack of clear institutional guidelines on WhatsApp for official health communication, leaving CHWs and supervisors to navigate complex grey zones of accountability and risk [43,44,45].

Critically, the uneven distribution of digital skills and access means that informal adaptation to WhatsApp can enable and exacerbate digital inequity. While younger and more technologically confident CHWs may thrive in WhatsApp-mediated workflows, older, rural, and digitally marginalized workers risk being left behind, further entrenching systemic inequities in program functioning and service quality [26,46,47]. These dynamics mirror broader global tensions around the promise and pitfalls of “smart” health systems, where the pace of technological change outstrips the capacity of systems to adapt inclusively and ethically [22,48,49].

Policy frameworks at both global and national levels increasingly call for user-centered, adaptive, and ethically robust approaches to digital health transformation [2,8,50,51,52,53]. Yet, there is a paucity of systematic, qualitative research capturing the lived realities of CHWs and supervisors regarding the role of WhatsApp in health and nutrition program delivery in aspirational districts as most published studies focus on formal mHealth solutions or quantitative program evaluations, with limited triangulation of perspectives across different cadres or critical analysis of how informal digital adaptation influences workflow, power relations, digital equity, and program outcomes [15,16,54].

This study aims to address these gaps by undertaking a COREQ (Consolidated Criteria for Reporting Qualitative Research; Tong, Sainsbury, & Craig, 2007 [55]) guided qualitative investigation into the use of WhatsApp among Community Health Workers (CHWs) and block-level officers in Muzaffarpur, Bihar [55]. As an aspirational district, Muzaffarpur exemplifies the persistent barriers and the potential of digital health in India’s most challenging environments, with lessons transferable to other low-resource LMIC settings. Grounded in the Health Belief Model (HBM) and borrowing a few constructs from the Technology Acceptance Model and the Unified Theory of Acceptance and Use of Technology, this research explores the drivers, benefits, risks, and consequences of WhatsApp integration at the community level.

The specific objectives were:

1. To examine the patterns and purposes of WhatsApp use among CHWs and block-level supervisors in managing community health and nutrition programs.
2. To identify the perceived benefits, barriers, and risks associated with WhatsApp-mediated communication, supervision, reporting, and peer support.
3. To assess how informal digital adaptation via WhatsApp influences workflow, power relations, digital equity, and program outcomes at the field level.

## 2. Materials and Methods

### 2.1. Study Design and Conceptual Framework

This qualitative study represents a prospectively registered, rigorously documented sub-study within a larger mixed-methods doctoral research protocol focused on digital health adoption among Community Health Workers (CHWs) and block-level officers in Muzaffarpur, Bihar, India. The qualitative component investigated WhatsApp’s operational realities, perceived benefits and barriers, and equity implications as an informal digital infrastructure for health and nutrition program delivery. The research followed the Consolidated Criteria for Reporting Qualitative Research (COREQ) 32-item checklist (Tong et al., 2007) [55].

The theoretical underpinnings mainly included the Health Belief Model (HBM) and integrating some key constructs of the Technology Acceptance Model (TAM), and the Unified Theory of Acceptance and Use of Technology (UTAUT) [56,57,58]. These frameworks guided the development of data collection instruments and the analytic approach, ensuring that individual, organizational, and technological determinants of digital adoption were comprehensively examined.

### 2.2. Study Setting and Rationale

Muzaffarpur, Bihar, is an officially designated Aspirational District under NITI Aayog’s programme. Bihar consistently ranks among India’s lower-performing states on several health and development indicators. Muzaffarpur is one of 13 Aspirational Districts in the state and among the most populous in the country. This makes it a relevant microcosm for examining the intersection of digital innovation, frontline program delivery, and equity [7,59,60,61,62,63,64,65,66,67]. The district’s diverse urban, peri-urban, and rural blocks provided an ideal setting for examining the intersection of digital innovation, frontline program delivery, and equity. Primary health and nutrition services in Muzaffarpur, like all other districts, are delivered through the Integrated Child Development Services (ICDS) and National Health Mission (NHM), with CHWs (AWWs, ASHAs, ANMs) and their supervisors (MOICs, CDPOs) at the operational core [10,68,69,70].

### 2.3. Study Registration, Approvals, and Ethical Conduct

The study was prospectively registered with the Clinical Trials Registry-India (CTRI/2023/04/051581) and received full ethical approval from the Institutional Ethics Committee of Manipal Academy of Higher Education, Manipal, India (IEC1:354/2022; IEC/MAHE/2023/145). Additional permissions were secured from the District Magistrate, Chief Medical Officer, and District Program Officer (ICDS) in Muzaffarpur. All study procedures conformed to the Declaration of Helsinki (2013 revision), national research ethics guidelines, and MDPI’s open data and ethical standards. Written informed consent was obtained from all participants, with explicit consent for audio recording and anonymized data publication.

### 2.4. Research Team and Reflexivity

The multidisciplinary research team consisted of the principal investigator (male, PhD scholar in public health, trained in qualitative research and digital health), a female field research assistant (to facilitate gender-sensitive interviewing), and senior public health and digital health supervisors and advisors. To minimize bias, no member of the research team had hierarchical authority over participants, and none of the members had any previous contact with any of the participants. Reflexivity was promoted through ongoing team debriefings, maintenance of field diaries, and analytic memos throughout the research process. The potential influence of the research team’s backgrounds, assumptions, and prior field experience was critically examined and transparently reported.

### 2.5. Sampling Strategy and Participant Recruitment

A purposive, maximum-variation sampling approach was employed to capture diverse perspectives across cadres, age groups, years of service, and settings [71,72,73]. The participant pool included frontline CHWs (AWWs, ASHAs, ANMs) and block-level supervisors (MOICs, CDPOs). We planned to conduct in-depth interviews with each cadre and focus group discussions stratified by urban/semi-urban and rural blocks to reflect contextual differences in infrastructure and workflow.

Four blocks were selected for diversity: Katra (rural, flood-prone), Paroo (rural), Musahari (urban), and Kanti (semi-urban). Inclusion criteria were active service, direct involvement in community health or nutrition programs, and regular smartphone use for work. Exclusion criteria were unwillingness/inability to provide informed consent or absence from field duties during data collection.

Participants were identified in collaboration with district health and ICDS officials and invited to participate through formal written communication and follow-up calls.

### 2.6. Data Collection Instruments and Pilot Testing

Semi-structured interview and FGD guides were developed in English, translated into Hindi, and back-translated to ensure conceptual fidelity. The guides were grounded in HBM, drew constructs from TAM and UTAUT, and included an updated literature review on digital health adoption. Domains covered patterns and frequency of WhatsApp use, perceived benefits and risks, digital literacy, administrative workload, peer learning, privacy, and the impact of digital communication on workflow and well-being. All instruments were pilot tested in a neighboring block not included in the final sample; revisions were made to maximize clarity, cultural relevance, and inclusivity.

### 2.7. Data Collection Procedures

IDIs and FGDs were conducted in-person between June and December 2023 at locations selected by participants for comfort and privacy (e.g., Anganwadi centers, Health and Wellness Centers, community halls). Each session began with a review of study aims, a detailed consent process, and assurances of confidentiality. Audio recordings were made with participant consent, and detailed field notes and analytic memos were recorded for each session. Interviews and FGDs lasted between 45 and 90 min. Data collection continued until thematic saturation was achieved.

Language used was participant-preferred (Hindi or local dialect), with bilingual researchers ensuring accurate translation. No financial incentives were provided; modest refreshments and local travel reimbursement were offered for FGD and IDI participation.

### 2.8. Transcription, Translation, and Data Anonymization

All audio recordings were transcribed verbatim in Hindi and translated line-by-line into English, retaining contextual Hindi terms or idioms in brackets. Bilingual researchers independently reviewed translations for fidelity. Personal identifiers, specific geographic locations, and organizational references were systematically removed or replaced with generic cadre/setting labels (e.g., [AWW, rural]) to ensure participant confidentiality and ethical compliance per COREQ.

### 2.9. Data Management and Analytical Approach

All data were managed in ATLAS.ti v23. We undertook reflexive thematic analysis using Braun and Clarke’s six-phase protocol [74]. The analytic steps included:

1. Familiarization: Repeated reading of transcripts, field notes, and analytic memos for immersion.
2. Initial Coding: The primary researcher coded transcripts, using constructs from the HBM, and selected elements from TAM and UTAUT to inform deductive codes, while allowing inductive codes to emerge from the data.
3. Theme Development: Codes grouped into themes and sub-themes through iterative discussion, memo-writing, and comparison across cadres and settings.
4. Theme Refinement: Collaborative naming and definition of themes under the guidance of supervisors and domain experts.
5. Synthesis: Selection of illustrative quotations and narrative integration of findings.
6. Reporting: Themes contextualized within current literature and programmatic context.

**Figure 1:**
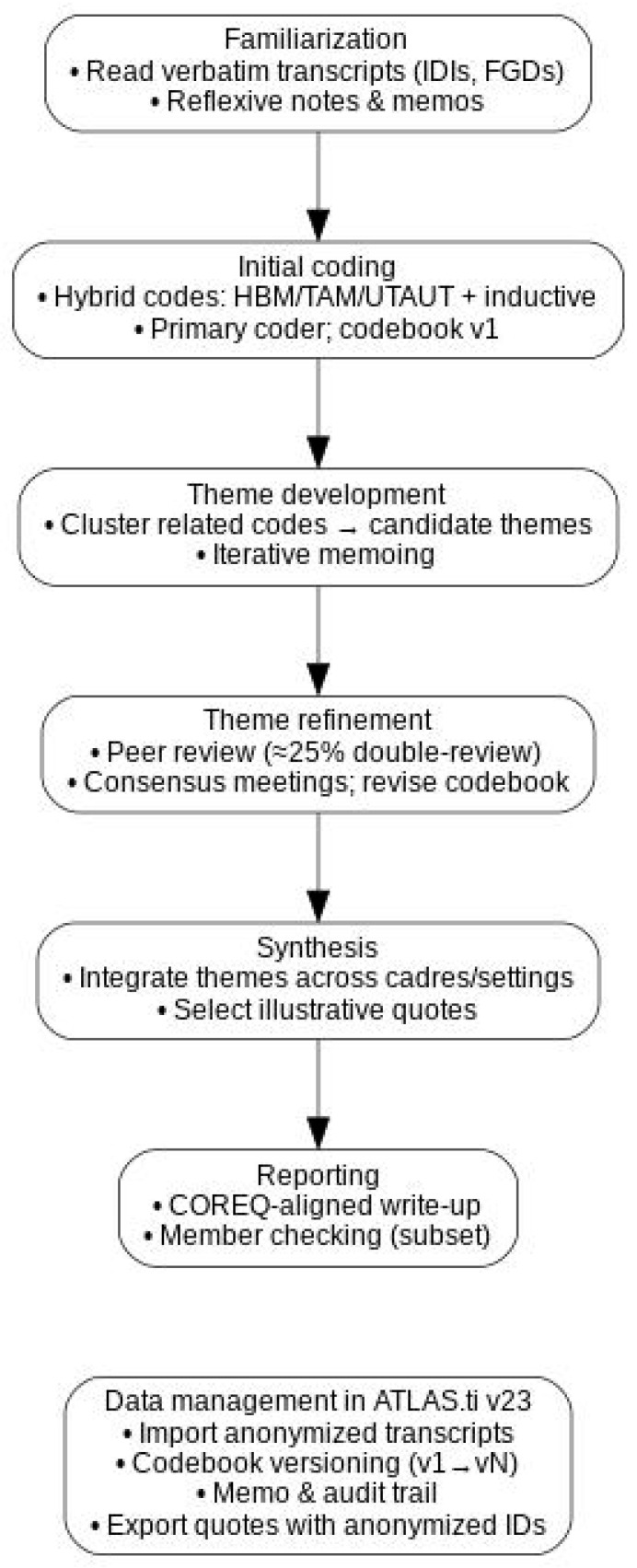
Reflexive thematic analysis workflow (Braun & Clarke, 2006 [74]) and data management in ATLAS.ti.

To enhance credibility, the supervisory team independently reviewed a subset (≈25%) of transcripts and the evolving codebook, with discrepancies resolved by consensus in scheduled analytic meetings. Informal member checking with a subset of participants was used to validate preliminary interpretations. The analytic workflow and data management sequence are summarized in Figure 1.

### 2.10. Trustworthiness and Data Sharing

Multiple strategies enhanced methodological rigor:

- Triangulation: Data sources (cadres, blocks), methods (IDIs, FGDs), and researchers.
- Reflexivity: Positionality diaries, team debriefings.
- Audit Trail: Full documentation of analytic decisions.
- Thick Description: Detailed context and participant characteristics to support transferability.
- Member Checking: Validation of interpretations with participants.

Data were stored securely on encrypted devices, accessible only to the research team. De-identified transcripts and analytic codebooks are available upon reasonable request from the corresponding author and are subject to ethical and institutional review.

## 3. Results

### 3.1. Participant Characteristics and Data Saturation

Between June and December 2023, thirty-two in-depth interviews (IDIs) and six focus group discussions (FGDs) were conducted, engaging a total of 81 participants: Community Health Workers (CHWs), including Anganwadi Workers (AWWs), Accredited Social Health Activists (ASHAs), Auxiliary Nurse Midwives (ANMs), and block-level officers (Medical Officers In-Charge (MOICs), Child Development Project Officers (CDPOs)). The study population reflected diverse cadres, geographies (urban, semi-urban, rural, and flood-prone blocks), age groups (mid-20s to late 50s), and years of professional experience (1–39 years). Thematic saturation was reached after 28 interviews and five FGDs, with subsequent data confirming the stability of codes and themes. Table 1 summarizes participant profiles, and Table 2 presents FGD group compositions.

Given persistent questions about whether WhatsApp reduces or increases work in practice, Table 4 contrasts how it relieved and added to workload across cadres and settings, with exemplar quotes.

### 3.2. Overview of WhatsApp’s Role

Reflexive thematic analysis identified five interrelated themes characterizing WhatsApp’s multifaceted role in health and nutrition program delivery: (1) WhatsApp as Informal Digital Backbone; (2) The Double-Edged Sword, Efficiency and Digital Burden; (3) Reshaping Supervision, Power, and Peer Support; (4) Digital Equity and Exclusion; and (5) Privacy, Well-being, and Policy Gaps. These themes are mapped to sub-themes with illustrative quotations in Table 3, while narrative detail follows.

#### 3.2.1. Theme 1: WhatsApp as the Informal Digital Backbone

Across all cadres and blocks, WhatsApp was unanimously described as the de facto digital infrastructure for daily work communication, rapid information dissemination, and peer problem-solving. Its adoption was both organic and near universal, transcending formal government platforms.

Reporting and Supervision: CHWs and block-level officers reported that WhatsApp groups facilitated the sharing of daily updates, government circulars, emergency alerts, and photographic evidence (e.g., immunization drives, nutrition events) in real time. An ANM (rural) shared, “Whatever instructions or updates come from the block, we get them instantly on WhatsApp—much before any written order or official call.” Supervisors similarly emphasized efficiency: “Supervision is now possible from anywhere. Earlier, we had to call each worker individually or wait for field visits, but now one message reaches everyone instantly” (CDPO, semi-urban, IDI).

Peer Problem-Solving: WhatsApp groups enabled CHWs to seek clarification, troubleshoot app issues, or coordinate field activities, especially when government apps (e.g., ANMOL, POSHAN Tracker) were inoperable due to network or server problems. “If one of us does not know how to fill out a new form or app entry, we ask in the WhatsApp group and someone always helps,” noted an AWW (urban, FGD). The COVID-19 pandemic further solidified WhatsApp’s status as a lifeline, providing real-time information flow when formal channels faltered.

#### 3.2.2. Theme 2: The Double-Edged Sword—Efficiency and Digital Burden

Information Overload and Fatigue: While WhatsApp streamlined communication, the volume and frequency of work-related messages created substantial digital fatigue. CHWs described receiving upwards of 100 messages daily, many outside of official work hours. “There are days I receive over a hundred messages. Even at night, supervisors expect replies. Sometimes it becomes impossible to rest,” reported an ASHA (urban, IDI). Late-night notifications, weekend demands, and expectations of immediate responses were everyday.

Administrative Duplication: WhatsApp reporting did not replace manual registers or mandatory data entry into official government apps. CHWs were routinely required to provide the same information in multiple formats: “We have to maintain both app entries and WhatsApp reporting double work, but the official portal is slow or not working, so we rely on WhatsApp to be safe” (AWW, rural, FGD). This “digital drudgery” was consistently cited as a source of frustration.

Work–Life Boundary Erosion: WhatsApp’s pervasiveness blurred the lines between professional and personal time. “Earlier, once the duty was over, I was free. Now, even at home, if a message comes, we must respond or give reasons” (ANM, flood-prone block, IDI). Many CHWs noted increased stress and disruption to family life. To clarify the workload dynamics, Table 4 contrasts how WhatsApp reduced and increased work across cadres and settings, with exemplar quotes.

#### 3.2.3. Theme 3: Reshaping Supervision, Power, and Peer Support

Enhanced Oversight and Micro-Management: WhatsApp enabled supervisors to practice near real-time oversight, requesting photographic proof of meetings or home visits, tracking attendance, and sending reminders. “I ask workers to send photos of meetings or field activities instantly—this is our proof and helps us track who is active” (MOIC, semi-urban, IDI).

Surveillance and Loss of Autonomy: For CHWs, the expectation of continuous digital visibility was described as “nigrani” (surveillance). “Sometimes it feels like we are always being watched, even for small delays. There is no privacy or trust left,” said an ASHA (rural, IDI). Features such as read receipts and last seen were used by supervisors as implicit monitoring tools.

Peer Learning and Solidarity: WhatsApp also fostered horizontal support, particularly in cadre-based groups. Tech-savvy CHWs often guided peers in troubleshooting app updates, sharing screenshots, and solving reporting challenges: “If one of us gets stuck, the group helps immediately. It is faster than calling the supervisor” (ANM, urban, FGD). The emotional support dimension was amplified during crises and heavy workloads.

#### 3.2.4. Theme 4: Digital Equity and Exclusion

Device and Connectivity Gaps: The assumption of universal WhatsApp access has masked significant disparities. Older, rural, or lower-income CHWs reported poor-quality phones, unreliable internet, and unaffordable data packs. “Some ASHAs still use basic phones. If there is an urgent WhatsApp message, they have to borrow someone else’s phone or miss out completely” (MOIC, rural, IDI).

Digital Literacy Divide: Younger, better-educated CHWs adapted more quickly to app and group use, while older or less-educated colleagues found group messages overwhelming or confusing. “Younger workers learn fast, but for us, these group messages are confusing,” shared an ANM (IDI).

Consequences of Digital Exclusion: Delayed responses or technical glitches often led to criticism or negative performance assessments. “He says, ‘Everyone else has sent it, what is your problem?’ He doesn’t understand that my phone’s memory is full and I can’t even download the message he sent,” explained an ASHA (IDI). Such incidents created anxiety and reinforced feelings of marginalization among digitally disadvantaged CHWs. Table 5 explains how the digital divide leads to a multidimensional divide.

#### 3.2.5. Theme 5: Privacy, Well-Being, and Policy Gaps

Unclear Boundaries and Risks: CHWs and officers described the absence of formal guidelines for WhatsApp use, especially for sharing sensitive data and photographs of beneficiaries. “Photos and personal details are shared without asking. There is no clarity on what is allowed or safe” (AWW, rural, IDI).

Emotional Well-Being: Many participants expressed stress and burnout arising from constant digital engagement and surveillance, coupled with the ambiguity of rules and lack of organizational support. “We need clear rules for when to respond, what can be shared, and who is responsible for data protection. Otherwise, WhatsApp is both a boon and a curse” (CDPO, urban, IDI).

Demand for Support: There was widespread consensus on the need for formal guidelines, provision of official devices, subsidized data, and digital literacy training, especially for older or rural workers. “We need phones, data, and training; otherwise, this system is only for those who already have these things” (ANM, semi-urban, FGD).

### 3.3. Cross-Cadre and Setting Comparisons

Rural community health workers faced more acute device and connectivity constraints, whereas urban groups described more peer-based troubleshooting, faster message relays, and easier coordination within cadre groups. Rural participants were more likely to rely on shared or family devices and reported delays when storage was full or data packs were unaffordable.

Across cadres, usage patterns diverged in line with role expectations. ASHAs leaned on WhatsApp for rapid clarifications, peer advice, and mobilization; AWWs used it chiefly for swift reporting, reminders, and submitting photographic proof when portals lagged; ANMs, who shoulder the largest volume of clinical and program data entry, reported the highest stress from duplicated reporting, constant visibility, and surveillance anxiety.

**Figure 2:**
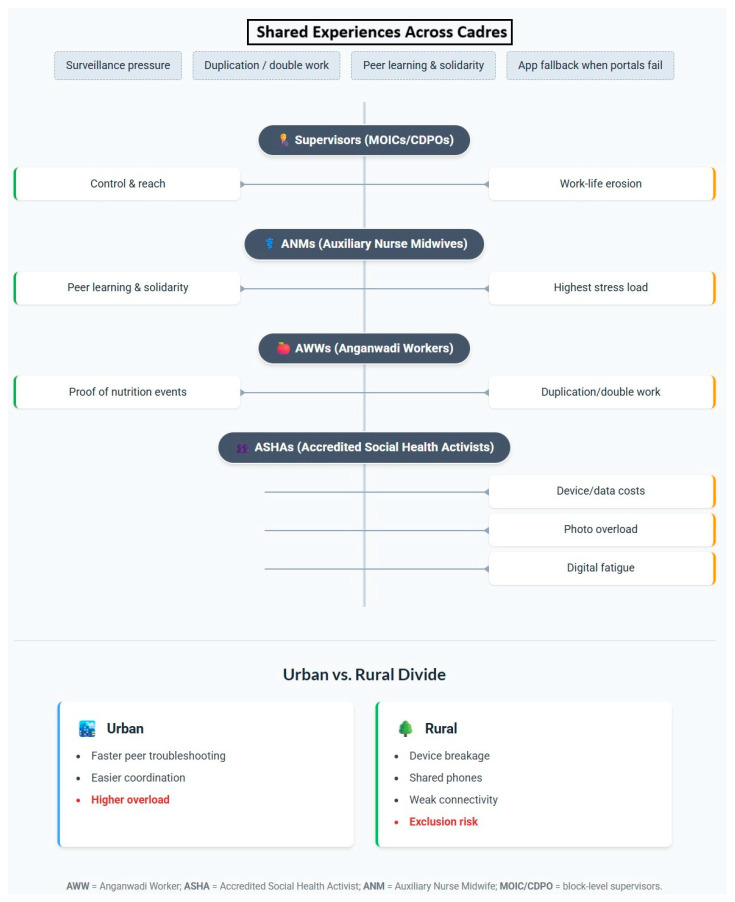
The Double-Edged Sword of WhatsApp: Shared and cadre-specific benefits and barriers among community health workers, with urban–rural digital divides highlighted.

Supervisors (MOICs, CDPOs) viewed WhatsApp as an enabler of transparency and reach. Many CHWs, however, experienced it as a mixed blessing—powerful for coordination and peer learning, yet intensifying after-hours expectations, administrative duplication, and perceived surveillance. Figure 2 summarizes these contrasts across cadres and settings, juxtaposing perceived benefits with the most frequently reported barriers.

## 4. Discussion

These qualitative insights, as part of a larger study conducted among Community Health Workers (CHWs) and block-level supervisors in an aspirational district of Bihar, India, provide robust empirical evidence on the operational role, impacts, and risks associated with the widespread informal adoption of WhatsApp for community health and nutrition program management. By engaging multiple cadres and incorporating both urban and rural perspectives, the research offers novel insights into the dynamics of digital health communication at the front lines, thereby advancing the understanding of informal digital infrastructures within public sector health systems in low- and middle-income countries (LMICs). This section critically interprets the results, situates them within the wider literature, discusses their broader significance, and addresses the study’s strengths and limitations.

### 4.1. Principal Findings and Their Context

Our study demonstrates that WhatsApp, though not an official government platform, has become the de facto digital backbone for day-to-day communication, reporting, supervision, and peer support across the health and nutrition workforce in an Indian aspirational district. The platform’s informality enables rapid information flow, real-time supervision, and agile peer learning outcomes, especially critical in resource-constrained, digitally fragmented environments [1,75]. These findings corroborate and extend recent research from India and other LMICs, highlighting the ascendancy of commercial messaging apps as pragmatic substitutes when robust formal mHealth systems are absent, insufficient, or technically unreliable [28,31,40,76].

By foregrounding voices from diverse cadres like AWWs, ASHAs, ANMs, and block officers, our analysis illuminates how WhatsApp’s utility is modulated by worker demographics, geographic context, and digital literacy, echoing global observations of a persistent “digital production gap” in health communication [26,54]. The narrative that emerges is one of both empowerment and exclusion: WhatsApp enables previously unimaginable coordination and supervision, but also intensifies digital workload, amplifies surveillance anxieties, and deepens pre-existing equity gaps, especially among older, rural, or resource-poor CHWs. These patterns mirror the workload and equity dynamics summarized in Table 4 and Table 5.

### 4.2. Comparison with Existing Literature

#### 4.2.1. WhatsApp as Informal Digital Infrastructure

Globally, the COVID-19 pandemic catalyzed the informal adoption of social media and commercial messaging platforms for health system resilience, particularly in LMICs [28,36,46]. Our results strongly support these trends, illustrating that in Indian field contexts, WhatsApp fills a crucial void left by the intermittent reliability of official platforms such as ANMOL or POSHAN Tracker. Similar findings have been reported from other countries like Ghana, Nigeria, Pakistan, and Brazil, where WhatsApp and associated groups allowed health workers to rapidly coordinate during outbreaks or policy shifts [31,46,76,77]. However, our work uniquely documents the persistent “parallel system” dynamic, where CHWs must duplicate reporting across WhatsApp and formal government apps, leading to administrative fatigue, a phenomenon less systematically analyzed in prior Indian studies.

#### 4.2.2. Peer Support, Supervision, and Surveillance

WhatsApp has emerged as a crucial platform for peer support among community health workers (CHWs), enabling rapid exchange of advice, encouragement, and best practices, especially where formal support is lacking. Studies from sub-Saharan Africa and South Asia highlight that WhatsApp groups build team morale, strengthen problem-solving capacity, and provide ongoing professional development for CHWs facing resource constraints [78,79,80].

However, these same digital tools increasingly facilitate supervisory surveillance, with supervisors using WhatsApp to monitor attendance, demand instant reports, and request photographic evidence of activities. This surveillance, while improving accountability, often intrudes into personal time and heightens stress, sometimes undermining trust and well-being among CHWs [38,79,80]. Thus, while WhatsApp fosters essential peer learning, it also creates new ethical and practical tensions in frontline health programs.

#### 4.2.3. Digital Equity, Literacy, and the Gendered Divide

A particularly salient contribution of this study is the documentation of how WhatsApp adoption, while nearly universal in intent, remains uneven in practice due to device access, data affordability, and digital literacy. This “digital inclusion-exclusion paradox” echoes recent conceptualizations of adverse digital incorporation, where the push to “go digital” risks amplifying social inequalities if not accompanied by systemic support [25,54]. Notably, our study found that rural, older, and lower-income CHWs were most vulnerable to exclusion and performance-related stress, with women facing additional challenges due to domestic responsibilities and limited digital confidence, findings resonant with data on digital gender divides [81,82,83,84].

#### 4.2.4. Cadre and Setting Contrasts: Interpretation and Program Relevance

Cadre-specific roles shaped how WhatsApp was experienced. ASHAs relied on cadre groups for just-in-time guidance and psychosocial support; AWWs emphasized reminders and photographic proof for activity reporting; and ANMs, who shoulder the largest clinical and program data workload, reported the most duplication and surveillance anxiety. Urban groups benefited from faster peer troubleshooting and device access, whereas rural and flood-prone blocks faced fragile phones, patchy networks, and out-of-pocket data costs that amplified digital exclusion. These contrasts argue against one-size digital policy; tiered supports should be tailored by cadre and context, with explicit relief of duplicative reporting and clear norms for after-hours communication.

#### 4.2.5. Policy Gaps, Privacy, and Well-Being

Our results underscore the lack of formal guidelines, training, and institutional safeguards around the use of WhatsApp for health work. The absence of data protection policies or clear protocols for sharing beneficiary information creates ethical risks and participant anxieties, echoing privacy concerns documented in digital health literature globally [44,50,52,85]. The findings thus highlight an urgent need for national and state policy attention on both the opportunities and risks of informal digital platforms in frontline health work.

### 4.3. Strengths of the Study

This study is notable for its comprehensive qualitative design, incorporating 32 in-depth interviews and six FGDs across all major CHW cadres and supervisory tiers in a challenging aspirational district setting. The use of both deductive (Health Belief Model-informed) and inductive thematic analysis, with rigorous saturation and double-coding, enhances the credibility, trustworthiness, and analytic depth of findings. Inclusion of block-level officers as well as frontline workers enables a rare “multi-level” perspective, strengthening the validity of cross-cadre and hierarchical interpretations. Further strengths include triangulation across cadres and settings, saturation across 32 IDIs and 6 FGDs, and analytic transparency using a reflexive thematic approach reported with COREQ.

In addition, the research directly addresses a critical evidence gap in Indian and LMIC literature: the real-world, day-to-day operationalization of WhatsApp as digital infrastructure, not merely as an add-on tool, but as the essential “glue” holding together fragmented health information and communication flows. By situating findings within both the national (e.g., Aspirational Districts Program, digital health strategies) and international (WHO Digital Health, SDGs) policy frameworks, the study supports both local and global relevance and transferability [7,50,53].

A further strength lies in the explicit reporting of ethical protocols, anonymization, and participant confidentiality, consistent with COREQ standards. The detailed documentation of device, data, and digital literacy barriers disaggregated by cadre and geography provides actionable insights for targeted policy and programmatic interventions.

### 4.4. Limitations and Mitigation Strategies

Several limitations warrant consideration. First, the study is confined to a single aspirational district in Bihar, which may limit generalizability to other contexts, especially states with differing health infrastructure or digital readiness. However, the district was selected due to its archetypal features (rural-urban mix, high vulnerability, multi-cadre CHW presence), enhancing the relevance to other high-burden settings in India, South Asia, and other LMICs.

Second, reliance on self-reported data through IDIs and FGDs may introduce recall bias or social desirability bias, particularly on sensitive topics like surveillance, stress, or digital exclusion. Careful interviewer training, gender-matched interviews, use of neutral prompts, and triangulation of narratives across cadres and methods mitigated this.

Third, the rapid evolution of digital platforms and policy guidelines means that WhatsApp usage patterns and regulatory frameworks may shift after data collection. To address this, findings are presented as a time-bound snapshot (June–December 2023), with recommendations for ongoing monitoring and adaptation in future research.

Fourth, although every effort was made to reach thematic saturation and include digitally excluded voices (e.g., those with basic phones or no WhatsApp access), some degree of selection bias is inevitable given the digital focus. This limitation is partially offset by purposive sampling, repeated member checking, and the inclusion of digitally fluent and digitally marginalized workers.

The qualitative nature of this study, while a strength in exploring depth and context, precludes the generation of quantifiable effect sizes or direct measurement of programmatic impacts. However, this is addressed by grounding the study in rigorous, internationally validated qualitative methods and by drawing on national program data for triangulation (Ministry of Women and Child Development, 2022 [10]). Finally, the study did not capture beneficiary perspectives, which limits inference about effects on client–provider trust and confidentiality. We also did not analyse WhatsApp message logs directly, relying on interview and FGD accounts; future mixed-methods work could triangulate logs with narratives.

### 4.5. Policy Implications and Recommendations

A dual approach is warranted. In the short term, programs should issue purpose-limited use protocols, consent language for images and identifiers, and response-time norms that protect work–life boundaries; in parallel, governments should supply reliable devices and data, redesign reporting to eliminate duplication with portals, and target training and supportive supervision to digitally excluded cadres.

(a) Institutionalization of Digital Health Guidelines: Ministries of Health and Women and Child Development, as well as state governments, should urgently develop and disseminate formal guidelines for the ethical, secure, and effective use of WhatsApp and similar platforms in health program delivery. This must include rules for data protection, explicit boundaries for out-of-hours communication, and protocols for sharing beneficiary information.
(b) Digital Equity Interventions: Provision of government-issued smartphones, subsidized data, and targeted digital literacy training are critical, particularly for older, rural, or marginalized CHWs. Gender-responsive approaches should be prioritized to address the intersectional barriers faced by women, as recommended by both Indian and global policy documents.
(c) Integration and Interoperability: Digital health strategy should focus on harmonizing formal government mHealth apps with existing informal platforms. Where duplication of reporting persists, urgent steps are needed to streamline workflows and reduce digital drudgery.
(d) Worker Well-Being and Organizational Support: Programs must address the psychosocial risks associated with constant connectivity and digital surveillance. This includes instituting “right to disconnect” policies, providing counseling or peer support, and recognizing the emotional labor of digital reporting.
(e) Research and Monitoring: Ongoing research should track the evolution of informal digital infrastructures, measure the impact of interventions, and include quantitative outcome measures alongside qualitative insights. National surveys and digital audits can help identify persistent or emerging gaps in digital equity and health system performance, especially the upcoming census in India, which could explore the use of digital applications and the use of digital health in different population groups, including the very large cadre of CHWs in India.

### 4.6. Directions for Future Research

Several avenues for future research emerge from this study. Longitudinal and mixed-methods research is needed to assess the long-term impacts of WhatsApp-based communication on CHW performance, program outcomes, and worker well-being. Experimental designs may help to evaluate the effectiveness of specific digital equity interventions (e.g., device provision, digital literacy workshops). Cross-country and cross-state comparisons will enrich understanding of context-specific versus generalizable findings.

Research must also expand to examine the perspectives of beneficiaries and community members, exploring how digital communication shapes service delivery and health outcomes at the last mile. Further, policy research should investigate the institutional enablers and barriers to the formal adoption or regulation of commercial messaging platforms within public sector health systems.

## 5. Conclusions

This study offers a timely, nuanced, and policy-relevant contribution to the literature on digital health integration in LMICs. By illuminating the complex, double-edged reality of WhatsApp’s informal adoption among CHWs and block-level officers, it demonstrates both the promise and peril of digital health innovations in resource-constrained settings. The evidence presented calls for a balanced, equity-oriented approach to digital health strategy, one that maximizes the empowering potential of informal platforms while proactively addressing the risks of exclusion, surveillance, and burnout. Policymakers, program managers, and researchers must work together to ensure that digital transformation in health serves as a bridge, not a barrier, to Universal Health Coverage and the Sustainable Development Goals.

## Figures and Tables

**Table 1:** Summary of Participant Characteristics.

Group	*n*	Age Range (Years)	Years of Service	Education	Annual Income (INR)
Block Officers	8	36–53	3–22	Bachelor’s/PG, MBBS	600,000–1,500,000
AWW (IDI)	8	25–54	4–28	Matric–MA	71,400
ASHA (IDI)	8	26–51	1–18	Matric–Graduate	26,000–60,000
ANM (IDI)	8	25–58	1–39	12th–Graduate	130,000–1,032,000

**Table 2:** FGD Group Profiles by Cadre and Location.

FGD Group	*n*	Age Range (Years)	Years of Service	Education Range	Annual Income (INR)
AWW Rural	7	39–50	16–20	Matric–Graduate	71,400
AWW Urban	8	36–52	1–24	Matric–MA	71,400
ASHA Rural	10	35–59	5–17	Matric–Graduate	30,000–41,000
ASHA Urban	8	27–46	2–14	Matric–Intermediate	36,000
ANM Semi-Urban	8	33–56	1–33	12th–Graduate	110,000–1,100,000
ANM Rural	8	27–58	1–30	12th–Graduate	131,000–200,000

**Table 3:** Thematic Structure: Themes, Sub-Themes, and Illustrative Quotes.

Theme	Sub-Themes	Representative Quotes
WhatsApp as Digital Backbone	Real-time reporting, peer problem-solving	“Instructions come instantly on WhatsApp—much before any written order.” (ANM, IDI)
Efficiency and Digital Burden	Information fatigue, work–life conflict, and duplication of reporting	“Supervisors expect replies even at night. Sometimes it becomes impossible to rest.” (ASHA, IDI); “We have to maintain both app entries and WhatsApp reporting—double work.” (AWW, FGD)
Reshaping Work and Peer Support	Digital surveillance, micro-management, peer learning	“I ask workers to send photos…this helps us track who is active.” (MOIC, IDI); “If we don’t know how to fill a new form, we ask in the WhatsApp group and someone always helps.” (AWW, FGD)
Digital Equity and Exclusion	Device/data gaps, literacy barriers, age/geography digital divide	“Some ASHAs still use basic phones…miss out completely.” (MOIC, IDI); “Younger workers learn fast, but for us, these group messages are confusing.” (ANM, IDI)
Privacy, Well-being, Policy Gaps	Unclear rules, stress, risk of data misuse	“Photos and personal details are shared without asking. There is no clarity on what is allowed or safe.” (AWW, IDI)

**Table 4:** The double-edged sword of workload: how WhatsApp reduced and increased work for CHWs and supervisors, with exemplar quotes.

Cadre/Setting	How WhatsApp Reduced Workload (Exemplar Quote)	How WhatsApp Increased Workload (Exemplar Quote)	Interpretation
AWW/Urban	“The biggest convenience is that we don’t have to travel for updates; everything gets done here itself.”	“It is compulsory to keep a register… But later, we are asked to click a picture as well.”	Digital submission and group updates reduce travel and delays; duplicative paper plus WhatsApp proof adds administrative load.
ASHA/Urban	“By sending it to the group, everyone gets the message.”	“Yesterday, while sitting on the terrace, I deleted 533 photos… Every day, 30–35 photos come in.”	Group broadcast speeds coordination; relentless photo traffic produces digital fatigue and crowds out recovery time.
ANM/Rural (flood-prone)	“If PHC sends an urgent letter or information, we receive it instantly on WhatsApp.”	“Even after reaching home… You are not free. You still have to send WhatsApp messages, SMS updates, and reports.”	Instant instructions reduce delays; after-hours expectations create invisible overtime and stress.
Supervision (MOIC/CDPO)/Semi-urban	“Supervision is now possible from anywhere… now one message reaches everyone instantly.” (CDPO, IDI)	“If we do any work, we have to take a photo and upload it.” (ASHA describing supervisory requirement)	Remote oversight accelerates supervision and reach; a “proof-by-photo” norm heightens perceived surveillance and privacy risks.

**Table 5:** Equity and resourcing constraints shaping WhatsApp use in the field.

Constraint Domain	Evidence from Transcripts (Cadre/Setting, Exemplar Quote)	Implications for Equity and Governance
Device quality and breakage	AWW, FGD (urban): “We were provided with government phones, but they are completely unusable now. We somehow manage to work using other people’s phones or by buying new phones ourselves.” AWW, IDI (urban): “I was given a government phone, but it got damaged, so I use my personal phone for work.”	Personal device dependence shifts costs and risks to workers; program functionality hinges on out-of-pocket replacements.
Connectivity and device performance	AWW, IDI (urban): “When the server is working fine, the process is quicker. [But] the server is also a problem for you? R: Yes.”	Poor connectivity undermines official apps, nudging routine work into WhatsApp as a fallback.
Data/SIM reimbursements	ASHA, FGD (urban): “It’s our personal phones, and we don’t even get money for recharges… There’s an option, but we haven’t received it till now.” AWW, FGD (urban): “The recharge costs ₹250, but we receive less than that… we don’t get it every month.”	Irregular or absent reimbursements create personal costs that disproportionately affect lower-paid cadres.
High media volume and storage burden	ASHA, FGD (urban): “So many photos come into the group… yesterday I deleted 533 photos. Every day, 30–35 photos come in.”	Constant multimedia inflow causes storage churn and digital fatigue, adding hidden time costs.
Official systems vs. WhatsApp workaround	ANM, IDI (urban): “If we have to send a report to the PHC, we send it via WhatsApp. If the report is short, we type it; otherwise, we write it down and then send a photo.”	WhatsApp becomes an informal reporting rail when portals lag, creating parallel data flows and governance gaps.
Phone performance degradation affecting official apps	ASHA, IDI (semi-urban): “The NCD app… has been hanging a lot on the government phone… Whenever we receive a lot of photos in the group, the phone freezes completely. When we try to open the NCD app, it does not respond… we have to manually delete everything from the gallery before we can use it again.”	Media-heavy WhatsApp use degrades device performance and disrupts mandated app use, risking data completeness.
Basic phones, digital literacy, and borrowing	AWW, IDI (urban): “Around 30 percent of them don’t know how to use a phone. They ask someone to do it for them, sometimes children or someone else helps them.” ASHA, IDI (urban): “It is my husband’s mobile phone, but I have taken it for my work.”	Skill and device gaps drive reliance on family devices and peer help, entrenching divides by age, gender, and education.
Privacy and security exposure	ASHA, IDI (urban): “During COVID, they used to ask for the public’s Aadhaar numbers… I told them, ‘If CDPO Ma’am says so, I will send it.’” AWW, FGD (urban): “Many workers have been scammed… Their bank accounts were emptied… After that, Madam sent a message in the group, saying, ‘If anyone receives a call from this number, do not answer it.’”	Ambiguous norms on sharing identifiers and widespread phishing highlight the need for clear consent and data-protection rules.

## Data Availability

The data presented in this study are available on request from the corresponding author due to restrictions imposed by the local administration and institutional requirements. The full anonymized transcripts cannot be made publicly available; however, codebooks and permission letters are provided as Supplementary Materials. Access to restricted data may be considered on reasonable request, subject to approval by the relevant authorities.

## Supplementary Materials

The following supporting information can be downloaded at: https://www.mdpi.com/article/10.3390/healthcare13172223/s1, File S1: COREQ 32-Item Checklist, File S2: Letter of Permission.

## Abbreviations

The following abbreviations are used in this manuscript:

ANM	Auxiliary Nurse Midwife
ANMOL	ANM Online
ASHA	Accredited Social Health Activist
AWW	Anganwadi Worker
CDPO	Child Development Project Officer
CHW	Community Health Worker
COREQ	Consolidated Criteria for Reporting Qualitative Research
CTRI	Clinical Trials Registry-India
FGD	Focus Group Discussion
HBM	Health Belief Model
HDI	Human Development Index
ICDS	Integrated Child Development Services
IDI	In-depth Interview
IEC	Institutional Ethics Committee
LMIC	Low- and Middle-Income Countries
MOIC	Medical Officer In-Charge
NHM	National Health Mission
SDGs	Sustainable Development Goals
TAM	Technology Acceptance Model
UTAUT	Unified Theory of Acceptance and Use of Technology
