# Supplementary material for: From Peer Support to Program Supervision: Qualitative Insights on WhatsApp as Informal Digital Infrastructure for Community Health Workers and Public Health Officers in an Indian High-Priority Aspirational District
Source: Healthcare (Basel). 2025 Sep 5;13(17):2223. doi: 10.3390/healthcare13172223 (PMC12428105; doi:10.3390/healthcare13172223)
Supplement: Supplementary file 1 [file healthcare-13-02223-s001.zip › Supplementary File S1 - COREQ 32-Item Checklist.pdf]

COREQ 32-Item Checklist

Manuscript Title:
From Peer Support to Program Supervision: Qualitative Insights on WhatsApp as Informal Digital Infrastructure for Community Health Workers and Public Health Officers in an Indian High-Priority Aspirational District

---

Domain / Item	Description	Manuscript Section	Page No.
**Domain 1: Research Team and Reflexivity**			
1. Interviewer/facilitator	PI (male PhD scholar) conducted IDIs/FGDs; supported by a female assistant for gender-sensitive facilitation	Methods – Research Team & Reflexivity	p. 6
2. Credentials	PI: PhD Scholar (Public Health, qualitative training); Co-authors: Professors, Social Scientist	Author info; Methods	pp. 1, 6
3. Occupation	PI: PhD Scholar; Co-authors: Professors / Associate Professor / Social Scientist	Author info	p. 1
4. Gender	Interviewer: Male; Assistant: Female	Methods – Reflexivity	p. 6
5. Experience and training	Training in qualitative methods and digital health; supervisory oversight	Methods – Reflexivity	p. 6
6. Relationship established	No prior relationship with participants	Methods – Reflexivity	p. 6
7. Participant knowledge of interviewer	Participants informed of aims, roles, confidentiality during consent	Methods – Data Collection Procedures	p. 8
8. Interviewer characteristics	Reflexivity ensured via debriefings, diaries, memos; no hierarchical authority	Methods – Reflexivity	p. 6
**Domain 2: Study Design**			
9. Methodological orientation	Reflexive thematic analysis (Braun & Clarke, 2006), COREQ guided	Methods – Analytical Approach	p. 9
10. Sampling	Purposive, maximum-variation sampling (cadre, geography, service years)	Methods – Sampling Strategy	p. 7
11. Method of approach	Formal letters + follow-up calls through district officials	Methods – Recruitment	p. 7
12. Sample size	32 IDIs, 6 FGDs; total n=81	Results – Participant	p. 11–12

		Characteristics; Tables 1–2	
13. Non-participation	Exclusion criteria reported; refusals not explicitly detailed	Methods – Recruitment	p. 7
14. Setting of data collection	Participant-preferred safe spaces (AWCs, HWCs, halls)	Methods – Data Collection Procedures	p. 8
15. Presence of non-participants	None; sessions conducted privately	Methods – Data Collection Procedures	p. 8
16. Description of sample	Cadre, block, age, education, years of service, income	Results – Tables 1 & 2	p. 11–12
17. Interview guide	Semi-structured guide, translated, back-translated, piloted	Methods – Data Collection Instruments	p. 8
18. Repeat interviews	No repeat interviews; one per participant	Methods – Data Collection Procedures	p. 8
19. Audio/visual recording	Audio-recorded with consent	Methods – Data Collection Procedures	p. 8
20. Field notes	Field notes and analytic memos maintained	Methods – Data Collection Procedures	p. 8
21. Duration	45–90 minutes per session	Methods – Data Collection Procedures	p. 8
22. Data saturation	Achieved after 28 IDIs & 5 FGDs; confirmed in Results	Results – Participant Characteristics	p. 11
23. Transcripts returned	No; informal member checking conducted	Methods – Analytical Approach	p. 9
**Domain 3: Analysis and Findings**			
24. Number of data coders	Primary coding by PI; subset (~25%) reviewed by supervisors/experts	Methods – Analytical Approach	p. 9
25. Description of coding tree	Themes, subthemes, quotes detailed; Table 3	Results – Table 3	p. 13–18
26. Derivation of themes	Hybrid deductive (HBM, TAM, UTAUT) + inductive	Methods – Analytical Approach	p. 9
27. Software	ATLAS.ti v23	Methods – Analytical Approach	p. 9

28. Participant checking	Informal member validation of findings	Methods – Analytical Approach	p. 9
29. Quotations presented	Illustrative quotes provided (Tables 3–5, narrative)	Results	p. 13–20
30. Data and findings consistent	Strong consistency; data support themes	Results; Discussion	p. 13–23
31. Clarity of major themes	Five clear themes; structured presentation; Figure 2	Results; Discussion	p. 13–23
32. Clarity of minor themes	Subthemes, cadre/setting contrasts in Tables 4–5	Results; Discussion	p. 13–23
