# Supplementary material for: From Peer Support to Program Supervision: Qualitative Insights on WhatsApp as Informal Digital Infrastructure for Community Health Workers and Public Health Officers in an Indian High-Priority Aspirational District
Source: Healthcare (Basel). 2025 Sep 5;13(17):2223. doi: 10.3390/healthcare13172223 (PMC12428105; doi:10.3390/healthcare13172223)
Supplement: Supplementary file 1 [file healthcare-13-02223-s001.zip › Supplementary File S2 - 5 Letter of Permission from DPO ICDS Muzaffarpur.PDF]

Letter of Permission

This is to certify that Mr. Anshuman who is a full-time Ph.D. research scholar at Manipal Academy of Higher Education (MAHE), Udupi, Karnataka, with his roll number 211100112, under the guidance of Dr. B Reshmi, Associate Dean & HOD DoHIM, MCHP, MAHE is being granted permission to conduct his research with the tentative title, "**Attitude to Adoption of Digital Health Technology among Front Line Workers and authorities in Public Health Setup: A Health Belief Model-based Qualitative Research**".

As stated by the applicant about the research being intended to provide insights on existing digital health platforms and might be able to help with future preparations, the respective officers of the relevant department are requested and expected to support for the study.

We wish him all the best with this research and offer our full support with the expectation that the research findings will be helpful for the department to strengthen the digital health systems.

Best Regards

[illegible]

DPO - ICDS
Muzaffarpur, Bihar
contact number - 9431005034
email Id - dpo.muz.051@gmail.com

जिला प्रोग्राम पदाधिकारी
मुजफ्फरपुर

समाहरणालय, मुजफ्फरपुर
(जिला प्रोग्राम कार्यालय I.C.D.S)

ज्ञापांक:- 2011 दिनांक:- 14/7/22

प्रतिलिपि : रश्मि बी० [MAHE-MCHP] को सूचनार्थ प्रेषित एवं निदेश दिया जाता है कि Research के फलाफल की प्रथम प्रति अधोहस्ताक्षरी को उपलब्ध कराना सुनिश्चित करेंगे।

[illegible]

जिला प्रोग्राम पदाधिकारी
मुजफ्फरपुर
